# Retraction: A model study of teaching method reform of computer laboratory course integrating internet of things technology

**DOI:** 10.1371/journal.pone.0318183

**Published:** 2025-01-23

**Authors:** The PLOS One Editors

The *PLOS One* Editors retract this article [1] because it was identified as one of a series of submissions for which we have concerns about potential manipulation of the publication process and authorship. In addition, we have concerns about the integrity of the peer review process. These concerns call into question the validity and provenance of the reported results. We regret that the issues were not identified prior to the article’s publication.

After a review of its electronic records, an official at Southern University and A&M College informed PLOS that there is no record of Marvin White being employed by its institution. Furthermore, the institution does not have a Department of Information Engineering.

This article [1] is similar to a withdrawn article [2] with the same title and author list.

All authors either did not respond directly or could not be reached.

## References

[1] Zhou X, Qian L, Aziz H, White M (2024) A model study of teaching method reform of computer laboratory course integrating internet of things technology. PLoS ONE 19(4): e0298534. 10.1371/journal.pone.0298534 38635843 PMC11025962

[2] Zhou X, Qian L, Aziz H, White M (2024). A Model Study of Teaching Method Reform of Computer Laboratory Course Integrating Internet of Things Technology. International Journal of Uncertainty, Fuzziness and Knowledge-Based Systems. 10.1142/S0218488524400142 PMC11025962 38635843

